# Revealing the bond exchange and network rearrangement mechanism in vitrimers

**DOI:** 10.1126/sciadv.aee9093

**Published:** 2026-04-17

**Authors:** Shinian Cheng, Lilliana Rey, Beibei Yao, Sophia K. Sabelja, Gaukhar Toleutay, Ivan Popov, Alexei P. Sokolov

**Affiliations:** ^1^ Department of Chemistry, University of Tennessee, Knoxville, TN 37996, USA.; ^2^ Chemical Sciences Division, Oak Ridge National Laboratory, Oak Ridge, TN 37831, USA.; ^3^ University of Tennessee-Oak Ridge Innovation Institute, University of Tennessee, Knoxville, TN 37996, USA.

## Abstract

Vitrimers combine thermosets stability with thermoplastic reprocessability, yet their rational design is hindered by an insufficient understanding of their viscoelasticity. We investigated the fundamental relationships among segmental dynamics, bond exchange kinetics, and network rearrangements in model poly(propylene glycol)–based vitrimers. Our results show that the bond rearrangement time (τ_*r*_) and its temperature dependence remain nearly unchanged at high temperatures, where their segmental relaxation time (τ_α_) differs by several orders. This indicates that rate-limited bond exchange kinetics controls high-temperature bond rearrangements largely independent of segmental dynamics. We further demonstrated that τ_*r*_ can exhibit either a stronger or weaker temperature dependence than τ_α_, depending on the observed temperature regime, addressing the long-standing puzzling of the relationship between these two processes. The observed results support the recently proposed framework where τ_*r*_ can be described as a two-step process, one controlled by bond exchange and another by segmental relaxation. However, simple additive description of characteristic timescales fails to provide consistent quantitative description.

## Teaser

A two-step mechanism drives bond rearrangements in vitrimers.

## INTRODUCTION

The escalating amounts of plastic pollution have resulted in notable ecological problems, underscoring the necessity of recyclable polymers design. Vitrimers have emerged as a leading candidate, redefining what cross-linked polymers can achieve (*1*–*3*). As a unique class of dynamic covalent networks, vitrimers undergo associative bond-exchange reactions without altering the overall cross-link density during rearrangements (*2*, *4*), a feature that imparts them with recyclability, self-healing, enhanced adhesive and shape memory properties, and exceptional toughness (*5*–*12*). However, the incorporation of dynamic covalent bonds introduces new parameters and additional relaxation processes spanning different length and timescales. This presents a key challenge to understand the fundamentals behind the relaxation dynamics and viscoelastic properties of vitrimers, strongly impeding their rational design with task-specific macroscopic properties.

The bond rearrangement time τ_*r*_ represents the key parameter in vitrimer dynamics, yet it remains the most challenging to elucidate. In traditional physical/transient networks (e.g., ionic or hydrogen bonded networks), τ_*r*_ always shows a stronger temperature dependence than structural (segmental) relaxation time τ_α_ because the activation energy barrier for bond rearrangements combines contributions from both local bond exchange and segmental mobility (*13*–*15*). Recent studies, however, revealed a more complex picture in vitrimers: In some systems, τ_*r*_ displays a weaker temperature dependence than τ_α_ (*16*–*18*), whereas, in others, it shows a stronger temperature dependence (*19*, *20*). Even more unexpectedly, certain vitrimers exhibit a clear transition from Arrhenius to super-Arrhenius behavior in τ_*r*_ upon cooling (*17*). These observations depart sharply from the classical theories of physical/transient polymer networks, indicating that fundamentally different molecular mechanisms govern the bond rearrangements in vitrimers.

Recent studies (*19*, *21*, *22*) proposed that the bond rearrangements in vitrimers involve two distinct events: (i) the diffusion of functional groups to collide with existing dynamic bonds, governed by segmental mobility; and (ii) a local chemical bond exchange, controlled by chemically specific reaction kinetics. Following this idea, τ_*r*_(*T*) is described as the sum of these two contributions (*19*, *23*)

$$\begin{array}{c} \tau_{r}\left(T\right)=A\text{exp}\left(\frac{E_{a}}{\mathrm{RT}}\right)+B\tau_{\alpha }\left(T\right)\\ =\tau_{0}\text{exp}\left(-\frac{∆S}{R}\right)\text{exp}\left(\frac{E_{a}}{\mathrm{RT}}\right)+\tau_{0}B\text{exp}\left(\frac{E_{a,\text{seg}}\left(T\right)}{\mathrm{RT}}\right) \end{array}$$ (1)

Here *E*_a,seg_ (*T*) is the temperature-dependent activation energy barrier for segmental motions, *E*_a_ is the temperature-independent enthalpic barrier for bond exchange, *R* is gas constant, *T* is the absolute temperature, the coefficient *B* reflects diffusion prefactor for the functional group and its potential multiple returns to the same bond before finding a different partner (*13*, *24*), τ_0_ ~ 10^−12^ to 10^−13^ s (*25*, *26*) is the typical elementary timescale of segmental relaxation prefactor, $A=\tau_{0}\text{exp}\left(-\frac{∆S}{R}\right)$ is the prefactor for time of the bond exchange process, and ∆S is the entropic barrier for bond exchange related to the chemical steric factor *p* = exp(Δ*S*/*R*) (*23*, *27*). While Eq. 1 seems to capture main features of τ_*r*_(*T*), detailed microscopic understanding of the prefactors *A* and *B* in this equation remains elusive (*22*, *28*). It is still unclear when τ_*r*_ has a stronger or weaker temperature dependence than τ_α_.

**Fig. 1.**
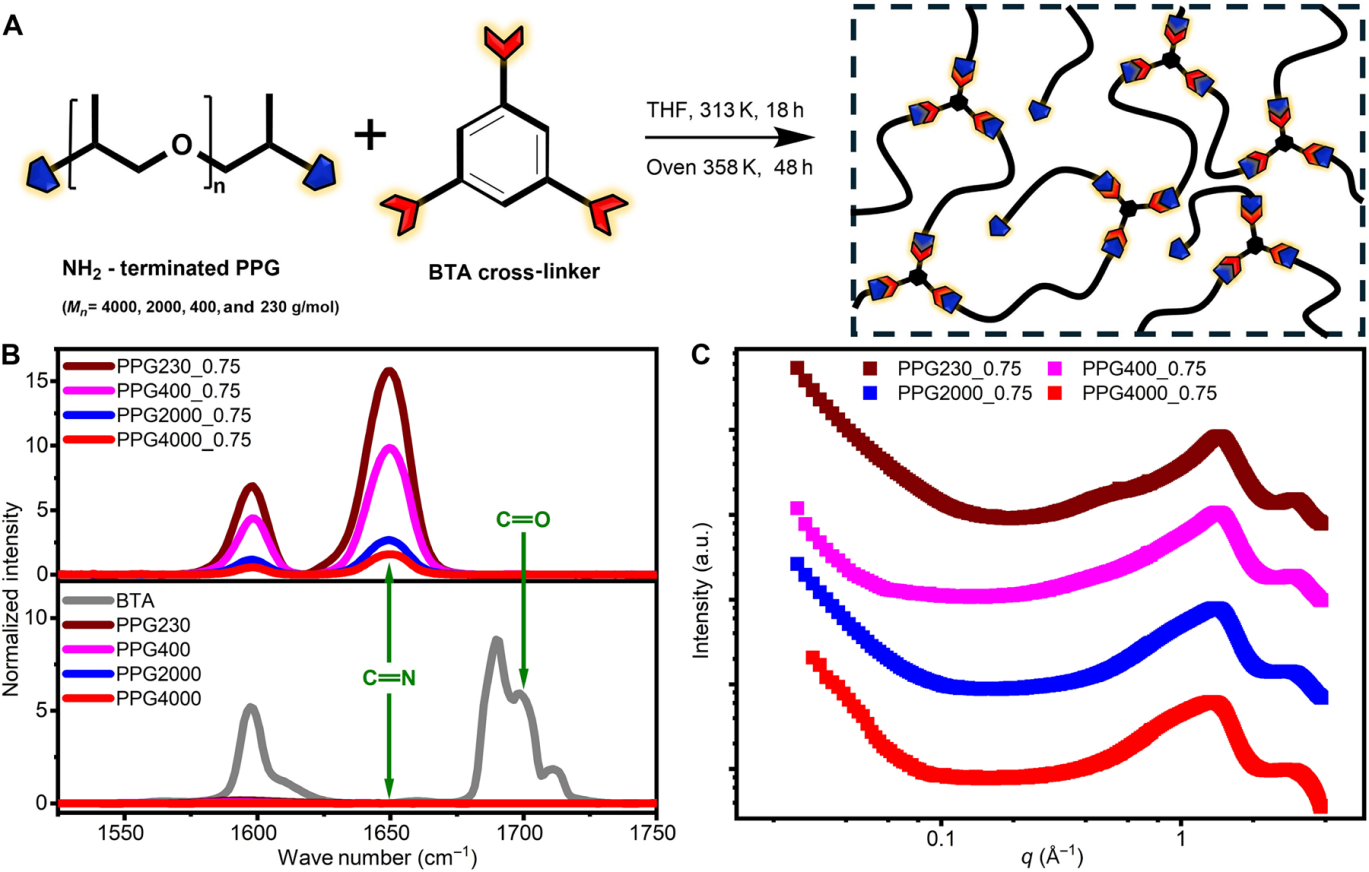
Molecular design and structure of vitrimers. (**A**) A schematic of the synthesis of vitrimer networks bearing imine bonds with a well-defined molecular weight between cross-linking. (**B**) Raman spectra confirming the formation of imine bonds in the synthesized samples. (**C**) X-ray scattering data confirming the absence of phase separation in all synthesized samples. a.u., arbitrary units.

In this work, we synthesized a series of model vitrimers bearing imine bonds by cross-linking amine-terminated telechelic poly(propylene glycol) (PPG) with benzene-1,3,5-tricarbaldehyde (BTA) (Fig. 1A). By varying PPG molecular weight (*M*_*n*_ = 230, 400, 2000, and 4000 g/mol), we studied the effect of *M*_*n*_ between cross-links on relaxation dynamics and viscoelastic properties in vitrimers through dielectric and rheological measurements in a wide frequency and temperature regime. Our results show that, as *M*_*n*_ decreases, the glass transition temperature (*T*_g_) of synthesized vitrimers strongly increases, which led to a substantial slowdown in the segmental relaxation time. Nevertheless, the bond rearrangement time τ_*r*_ and its temperature dependence remain nearly unchanged at high temperatures, highlighting the negligible influence of segmental mobility on bond rearrangement process in this high-temperature regime. Furthermore, τ_*r*_ exhibits a crossover from Arrhenius to super-Arrhenius behavior upon cooling at *T*_g_/*T* ~ 0.7. Our results also revealed that τ_*r*_ can exhibit either stronger or weaker temperature dependence than τ_α_, depending on the distance of experimental temperature from *T*_g_. This resolves the long-standing puzzle of why some vitrimers exhibit a stronger temperature dependence of τ_*r*_ than τ_α_, while others exhibit a weaker temperature dependence. Together, our findings support the recently proposed picture (*19*, *21*, *22*) that the bond rearrangement time in vitrimers can be approximated as a sum of the bond exchange and segmental relaxation time. However, this analysis also revealed very large values of the parameter *B* ~ 10^7^ to 10^9^ in Eq. 1, and a microscopic understanding of this value remains a puzzle.

## RESULTS

Model vitrimers consist of PPG chains with well-defined molecular weights (i.e., *M*_*n*_ = 230, 400, 2000, and 4000 g/mol) connected by BTA cross-linkers forming imine bonds with PPG chain ends (Fig. 1A). The networks were designed to have 25% excess of unreacted ─NH_2_ end groups to enable the transimination bond rearrangement mechanism because the often claimed metathesis mechanism of imine bonds was shown to be inefficient without catalysts (*20*, *23*). Raman spectroscopy confirmed and quantified the formation of imine bonds in the synthesized samples. Compared to the PPG precursor and BTA cross-linker, Raman spectra of synthesized samples show two clear features: (i) the occurrence of an additional peak near 1649 cm^−1^, demonstrating the formation of C═N bond and (ii) the complete disappearance of peaks around 1700 cm^−1^, indicating no left aldehyde groups in the synthesized samples (Fig. 1B). After normalization of the Raman spectra to the peak at 1099 cm^−1^ (representing the C─O bonds in the PPG backbone), a clear increase in the peak area at 1649 cm^−1^ was observed as the molecular weight between cross-linking junctions decreases (Fig. 1B), demonstrating an increase in cross-linking density. The small-angle x-ray scattering (SAXS) data did not show any features at lower *q* range (Fig. 1C), demonstrating the absence of nanoscale phase separations in the studied vitrimers.

**Fig. 2.**
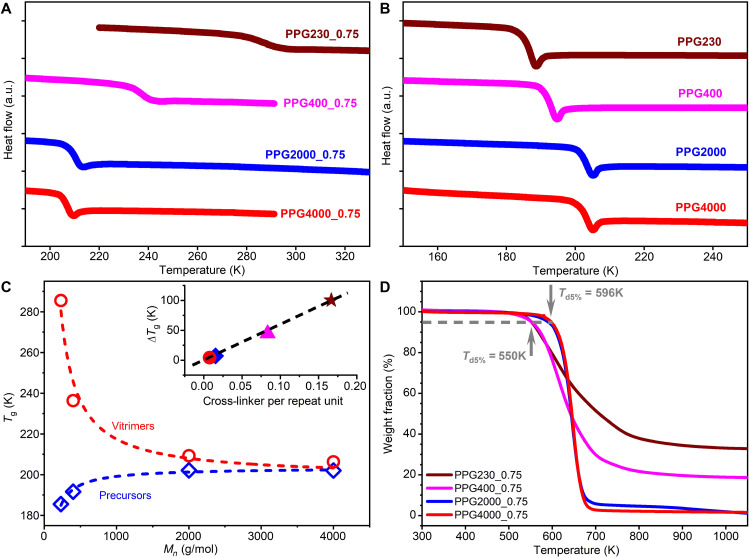
Thermal properties of studied vitrimers and their corresponding polymer precursors. DSC heating curves for (**A**) PPG-based vitrimers and (**B**) PPG polymer precursors with different molecular weights. a.u., arbitrary units. (**C**) The evolution of glass transition temperature with polymer molecular weight in both vitrimers and precursors. Dashed lines are fits to Flory-Fox equation. Inset: Glass transition temperature difference as a function of cross-linker density. (**D**) TGA thermograms of studied vitrimers.

We used differential scanning calorimetry (DSC) and thermogravimetric analysis (TGA) to study the thermal properties of the synthesized vitrimers. The DSC data of both vitrimers (Fig. 2A) and polymer precursors (Fig. 2B) exhibit a clear endothermic step upon heating, which is associated with their glass transition processes. For synthesized vitrimers, the endothermic step shifts to lower temperature as *M*_*n*_ increases, suggesting a decrease in *T*_g_. Similar observation was reported in vitrimers with boronic ester cross-links synthesized by reacting telechelic alkane diols and boric acid (*29*). These results are consistent with the well-known decrease in *T*_g_ with decrease in cross-linking density. In contrast, increasing the *M*_*n*_ of PPG precursors leads to the usual increase in *T*_g_ for chains with weak end groups interactions (Fig. 2B). Therefore, the glass transition temperature difference Δ*T*_g_ between vitrimers and their corresponding polymer precursors decreases as *M*_*n*_ increases (Fig. 2C). Furthermore, a linear correlation between Δ*T*_g_ and cross-linker per repeat unit was found in studied samples as shown in the inset of Fig. 2C. This result is consistent with the recently developed statistical mechanical theory (*30*) that predicts a linear dependence between Δ*T*_g_ and the sticker fraction for associating polymer melts. Furthermore, the synthesized vitrimers show good thermal stability with 5% decomposition temperature *T*_d5%_ = 550 K for PPG230/400_0.75 and *T*_d5%_ = 596 K for PPG2000/4000_0.75.

**Fig. 3.**
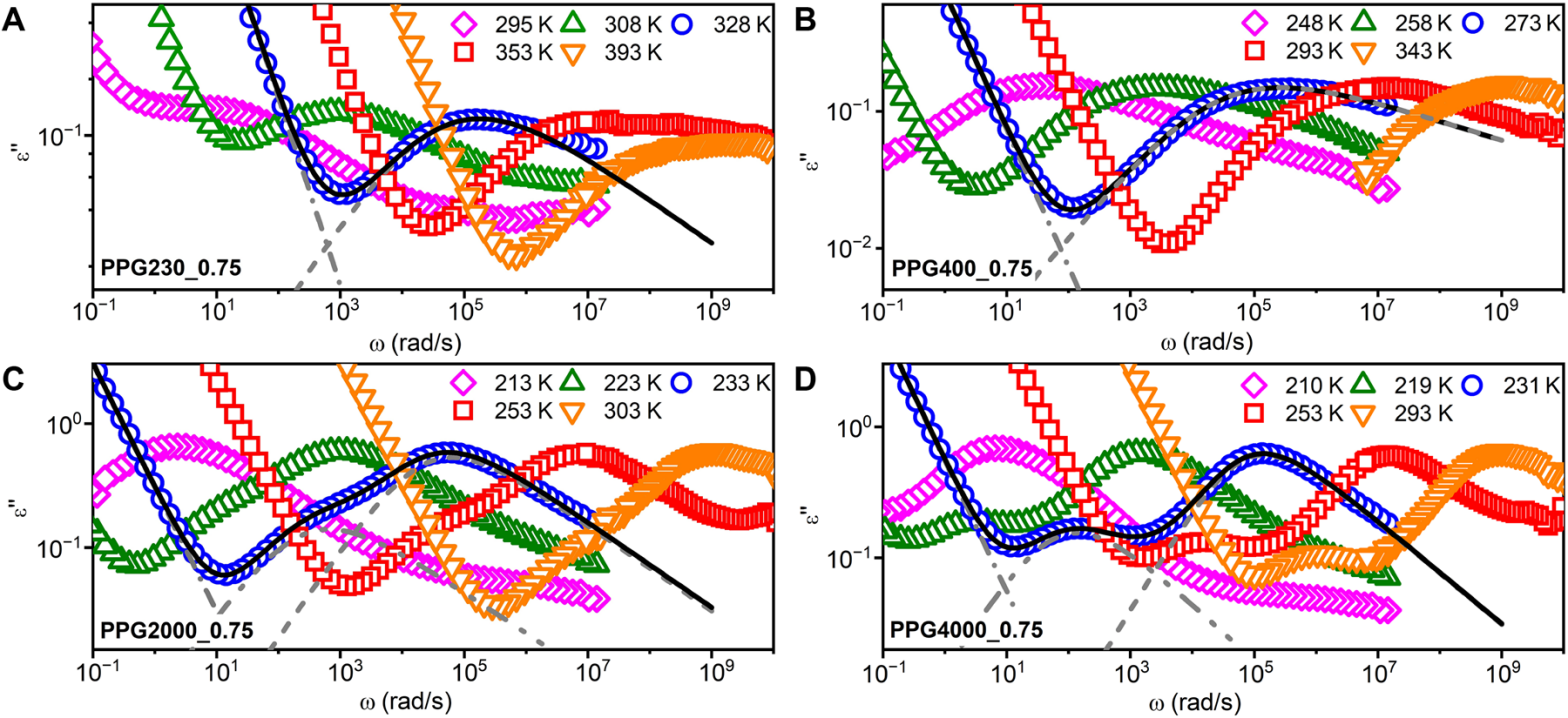
The representative dielectric loss spectra of studied vitrimers at selected temperatures. (**A**) PPG230_0.75, (**B**) PPG400_0.75, (**C**) PPG2000_0.75, and (**D**) PPG4000_0.75. The lines in each panel show the representative fit of the spectra to Eq. 2. The gray dash-dot, dash-dot-dot, and dashed lines present the dc-conductivity contribution, the chain relaxation mode, and segmental relaxation mode, respectively. The black line is the sum of all contributions to the dielectric spectra.

Two well-separated relaxation modes were observed in dielectric permittivity spectra of PPG2000_0.75 and PPG4000_0.75 (Fig. 3, C and D): The high-frequency peak is related to the segmental dynamics and low-frequency one to the end-to-end chain dynamics as PPG has an accumulated dipole moment along the chains (*31*). For PPG230_0.75 and PPG400_0.75, the dielectric permittivity $\varepsilon^{\prime \prime }\left(\omega \right)$ in the examined frequency window does not show well-separated chain modes and, instead, exhibits one broad relaxation peak that represents the segmental dynamics (Fig. 3, A and B). To extract the relaxation times, the dielectric spectra were analyzed using two Havriliak-Negami (HN) functions (*32*)

$$\varepsilon^{*}\left(\omega \right)=\varepsilon_{\infty }+\sum_{j=\alpha ,n}\frac{\Delta \varepsilon_{j}}{{[1+{\left(i\omega \tau_{\text{HN},j}\right)}^{\beta_{j}}]}^{\gamma_{j}}}+\frac{\sigma_{\text{DC}}}{i\omega \varepsilon_{0}}+a\omega^{-b}$$ (2)

where *i* is the imaginary unit, $\varepsilon^{*}$ is the complex permittivity, $\varepsilon_{\infty }$ and $\varepsilon_{0}$ are the dielectric constants at the infinitely high frequency and the vacuum permittivity, and ω is the angular frequency. Additionally, τ_HN,*j*_, ${\Delta \varepsilon }_{j}$, β*_j_*, and γ*_j_* are the characteristic HN time, dielectric amplitude, and shape parameters of the segmental (*j* = α) and chain (*j* = *n*) relaxation processes, respectively. The final two terms represent dc conductivity, $\sigma_{\text{DC}}$, and electrode polarization described by two constants *a* and *b*. To fit the dielectric spectra of PPG230_0.75 and PPG400_0.75, which exhibits only one relaxation peak, the relaxation strength of the second HN functions was set to be zero. The characteristic relaxation time τ that corresponds to the maximum position of the dielectric loss peak can be obtained from the characteristic HN time (*32*)

$$\tau_{j}=\tau_{\text{HN},j}{\left[\text{sin}\frac{\beta_{j}\pi }{2+2\gamma_{j}}\right]}^{-1/\beta_{j}}{\left[\text{sin}\frac{\beta_{j}\gamma_{j}\pi }{2+2\gamma_{j}}\right]}^{1/\beta_{j}},\text{with}\;j=\alpha \;\text{or}\;n$$ (3)

**Fig. 4.**
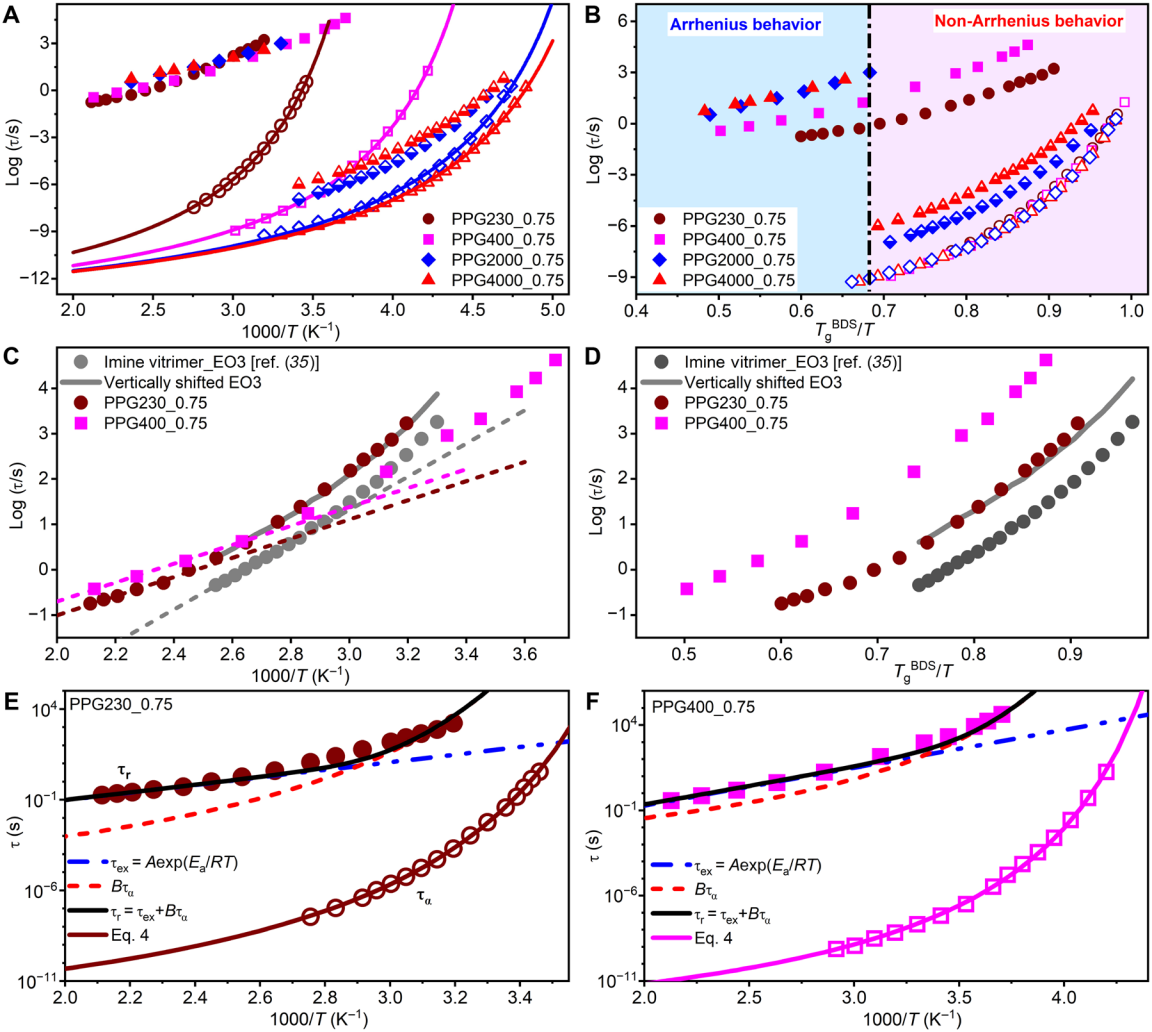
Temperature-dependent characteristic timescales of studied vitrimers. Segmental/structural relaxation time τ_α_ (open), chain relaxation time τ_*n*_ (half solid), and bond rearrangement time τ_*r*_ (solid) as a function of (**A**) 1000/*T* and (**B**) *T*_g_^BDS^/*T*. The solid lines present the fits of τ_α_ to Eq. 4 with fixed $\tau_{0}=10^{-13}$ s. The determined *D* and *T*_0_ are displayed in Table 1. The black dashed dot line in (B) indicates the possible boundary of the crossover in τ_*r*_(*T*) from Arrhenius to non-Arrhenius. The comparison of τ_*r*_(*T*) data between PPG230_0.75, PPG400_0.75, and imine-based vitrimer with EO3 cross-linker from (*35*) plotted versus (**C**) 1000/*T* and (**D**) *T*_g_^BDS^/*T*. The dashed lines in (C) display the Arrhenius fitting of τ_*r*_(*T*) data in the high-*T* region. The solid gray curves in (C) and (D) are vertically shifted EO3 data to match with PPG230_0.75. The black solid line displays the best fit of experimental τ_*r*_(*T*) data to Eq. 1 for (**E**) PPG230_0.75 and (**F**) PPG400_0.75. The determined *A* and *B* are shown in Table 1. In this fitting, τ_α_(*T*) was described using Eq. 4 (solid lines) with $\tau_{0}=10^{-13}$ s and the determined *D* and *T*_0_ in Table 1.

The determined segmental/structural relaxation time τ_α_ and chain relaxation time τ_*n*_ are plotted as a function of temperature in Fig. 4.

**Fig. 5.**
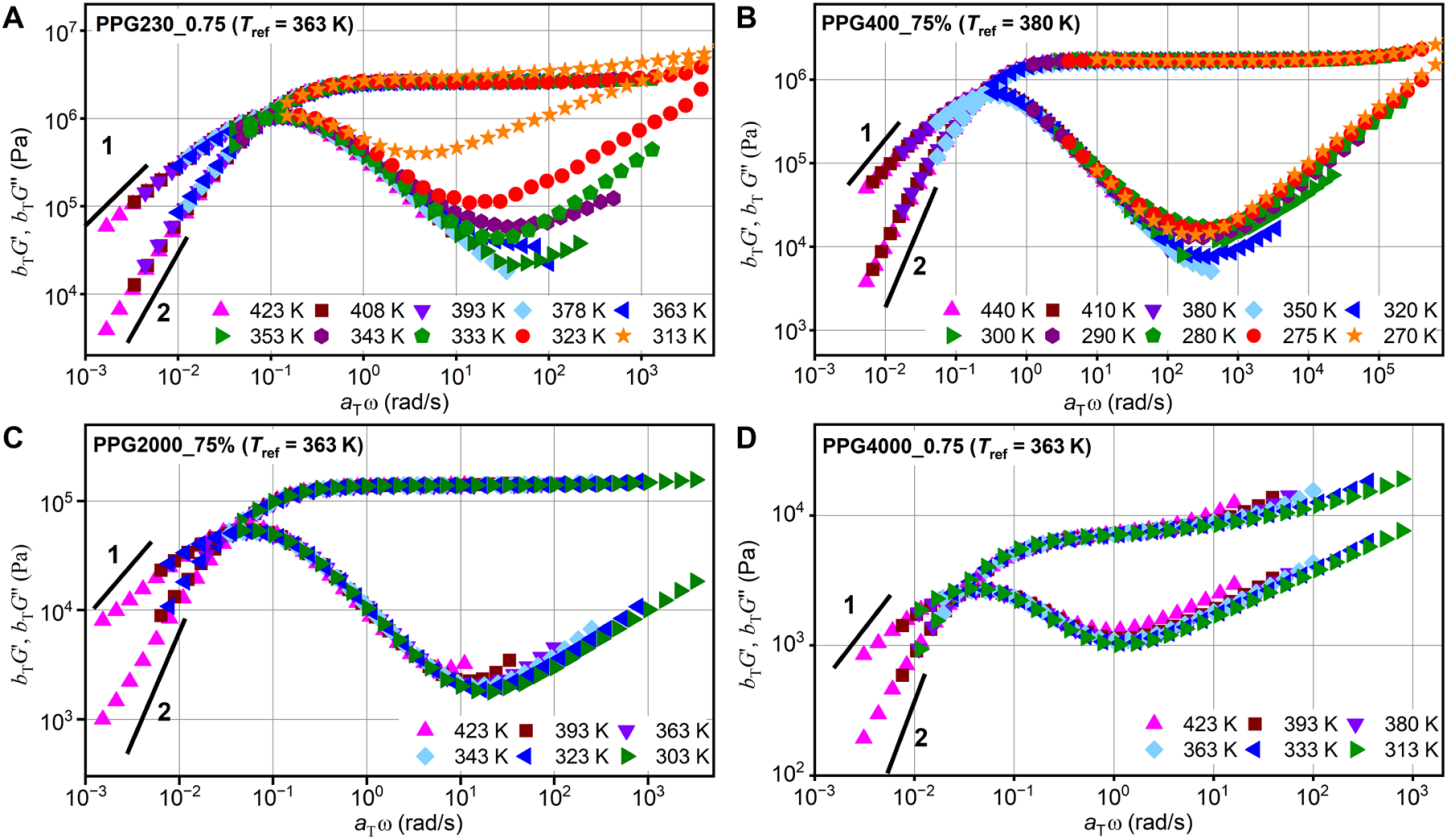
Rheology master curves of shear modulus for studied vitrimers. (**A**) PPG230_0.75, (**B**) PPG400_0.75, (**C**) PPG2000_0.75, and (**D**) PPG4000_0.75. The solid lines in each panel represent the theoretical power law in the terminal flow regime as a comparison to the experimental data.

We studied the linear viscoelasticity of obtained vitrimers using small-amplitude oscillatory shear (SAOS) rheological measurements. The shear modulus master curves of $G^{\prime }\left(\omega \right)$ and $G^{\prime \prime }\left(\omega \right)$ (Fig. 5) were constructed by, first, using a horizontal shifting of the phase angle spectra and, then, simultaneously vertically shifting both the storage and loss shear moduli until aligned with the respective previous temperature scan. Although time-temperature superposition does not work in this case (as evident, e.g., from changes around the minimum in $G^{\prime \prime }\left(\omega \right)$; Fig. 5), the obtained master curves provide a good qualitative overview of the viscoelastic properties in the studied vitrimers. The shear modulus spectra show the typical linear viscoelastic response of vitrimers under oscillatory shear with a well-defined frequency-independent rubbery plateau whose magnitude is defined by the concentration of imine cross-links. A clear drop in the storage modulus $G^{\prime }\left(\omega \right)$ at low frequencies and a corresponding relaxation peak observed in the loss modulus $G^{\prime \prime }\left(\omega \right)$ represent the stress relaxation of the polymer networks due to the bond rearrangement process. Following the traditional rheological approach based on classical Maxwell model (*33*), we obtained the bond rearrangement time $\tau_{r}=1/\omega_{\text{cross}}$ (Fig. 4), where ω_cross_ is the angular frequency of the $G^{\prime }\left(\omega \right)$ and $G^{\prime \prime }\left(\omega \right)$ crossover. At temperatures where the crossover between $G^{\prime }\left(\omega \right)$ and $G^{\prime \prime }\left(\omega \right)$ is outside the experimental frequency window, the horizontal shift factor was used to estimate $\tau_{r}$.

## DISCUSSION

Segmental relaxation time τ_α_ of the studied vitrimers exhibit super-Arrhenius temperature dependence in the studied experimental window and is analyzed using the Vogel-Fulcher-Tamman equation (Fig. 4A)

$$\tau_{\alpha }\left(T\right)=\tau_{0}\text{exp}\left(\frac{E_{a,\text{seg}}\left(T\right)}{\mathrm{RT}}\right)=\tau_{0}\text{exp}\left(\frac{DT_{0}}{T-T_{0}}\right)$$ (4)

where τ_0_ is fixed at $\tau_{0}=10^{-13}s$ and *D* and *T*_0_ are fitting parameters. The glass transition temperature *T*_g_^BDS^ estimated using τ_α_ (*T*_g_^BDS^) = 100 s criterion is consistent with *T*_g_^DSC^ (Table 1) from calorimetric measurements for all studied vitrimers. Although τ_α_ strongly slows down as *M*_*n*_ decreases (i.e., cross-link density increases), its temperature variation shows no dependence on *M*_*n*_ or cross-link density after accounting for the changes in *T*_g_ (Fig. 4B), suggesting no change in the dynamic fragility of studied vitrimers. These results indicate that, while increasing cross-link density strongly slows down the local segmental motions, it has negligible effect on their temperature dependences after scaling to *T*_g_, which is consistent with the prediction from Schweizer’s statistical mechanical theory originally developed for associative polymers (*30*).

**Table 1.** Parameters of the studied vitrimers. NA, not available.

	*T*_g_^DSC^ (K)	*T*_g_^BDS^ (K)	*E*_a_ (kJ mol^−1^)	*A/*s	Δ*S* (J K^−1^ mol^−1^)	*B*	*D*	*T*_0_ (K)
PPG230_0.75	285 ± 1	284 ± 1	41 ± 3	6.3 × 10^−6^	−149.3	2 × 10^7^	6.8 ± 0.1	237.4 ± 0.3
PPG400_0.75	237 ± 1	236 ± 1	43 ± 2	6.8 × 10^−6^	−149.9	5 × 10^9^	6.4 ± 0.1	199.3 ± 0.5
PPG2000_0.75	209 ± 1	207 ± 1	44 ± 2	1.2 × 10^−5^	−154.7	NA	6.5 ± 0.1	173.7 ± 0.6
PPG4000_0.75	206 ± 1	203 ± 1	42 ± 1	3.2 × 10^−5^	−162.8	NA	6.5 ± 0.1	170.3 ± 0.4

One interesting finding in Fig. 4A is that $\tau_{r}$ of the studied vitrimers at high temperatures remains almost unchanged although $\tau_{\alpha }$ slows down notably as *M*_*n*_ decreases. This demonstrates the decoupling of bond rearrangement process from segmental relaxation in studied vitrimers at high temperatures. Furthermore, the analysis of $\tau_{r}$ in the high-*T* regime gives almost the same activation energy *E*_a_ ~ 43 ± 2 kJ mol^−1^ and prefactor *A* ~ 10^−5^ s in all studied vitrimers (Table 1), suggesting that $\tau_{r}$ of the studied vitrimers is almost independent of segmental relaxation in the high-*T* regime, which is consistent with recent simulations (*22*). It is worth noting that the determined *E*_a_ ~ 43 ± 2 kJ mol^−1^ and *A* ~ 10^−5^ s in studied vitrimers are consistent with those reported in other imine-bonded vitrimers (*20*, *23*). Yet, the prefactor *A* increases slightly with *M*_*n*_ (Table 1). We emphasize that prefactor *A* depends not only on the chemically specific bond switching mechanism but also on the probability of a free sticker to find a bond for switching. In our work, the slight increase in the prefactor *A* in higher *M*_*n*_ vitrimers is due to the lower probability of a free ─NH_2_ end group to find an imine bond. The average distance *R* between imine bonds increases as $R\propto M_{n}^{1/3}$, while concentration of free amine groups decreases $\propto M_{n}^{-1}$. This reduces the probability for their collision, leading to slower bond exchange time and therefore larger prefactor *A* in systems with higher $M_{n}$. Another interesting finding is the change in $\tau_{r}$ from Arrhenius to super-Arrhenius temperature dependence upon cooling for PPG230_0.75 and PPG400_0.75 (Fig. 4A), highlighting that different mechanisms control the bond rearrangements at high (far above *T*_g_) and low (close to *T*_g_) temperatures.

The recently proposed idea (*19*, *21*) describes bond rearrangements in vitrimers as a two-step process involving chemical exchange and diffusion. In that case, bond rearrangement time presented as a sum of the timescales of bond exchange and segmental diffusion (Eq. 1) qualitatively explains the observed results of $\tau_{r}\left(T\right)$ (Fig. 4). At high temperatures far above *T*_g_, the experimental $\tau_{r}$ shows no remarkable dependence on segmental dynamics (Fig. 4A), suggesting that the bond exchange controls the bond rearrangements according to Eq. 1, namely, $\tau_{r}\approx \tau_{\text{ex}}=A\text{exp}\left(\frac{E_{a}}{\mathrm{RT}}\right)$, here $\tau_{\text{ex}}$ denoting a local bond exchange time. This relationship has been demonstrated recently using coarse-grained computer simulations where *E*_a_ of the bond exchange was tuned by model parameters (*22*). It explains why all the studied vitrimers exhibit almost the same τ_*r*_(*T*) at the high-*T* regime, although their segmental dynamics slow down tremendously as *M*_*n*_ decreases. As suggested recently, the super slow associative bond rearrangements even at very high temperatures originate from the extremely large entropic barrier (*23*) or low chemical steric factor (*34*) for associative dynamic covalent bond exchange. Because PPG230_0.75 and PPG400_0.75 show much higher *T*_g_, especially for PPG230_0.75, compared to PPG2000_0.75 and PPG4000_0.75 (Table 1), the experimental temperature window of $\tau_{r}$ becomes closer to the *T*_g_ of PPG230_0.75 and PPG400_0.75. At certain lower temperatures, the bond rearrangements become more sensitive to segmental relaxation, namely, *B*τ_α_ becomes comparable to τ_ex_. In this case, the contribution from segmental dynamics to bond rearrangement process cannot be negligible, and, therefore, $\tau_{r}\left(T\right)$ exhibits a crossover from Arrhenius to non-Arrhenius behavior in PPG230_0.75 and PPG400_0.75 at *T*_g_^BDS^/*T* ~ 0.7 (Fig. 4B). At higher temperatures, $\tau_{r}\left(T\right)$ exhibit a well-defined Arrhenius behavior with activation energy essentially independent on chain length. However, we do not expect that the value *T*_g_^BDS^/*T* ~ 0.7 for crossover regime is general; it should depend on specific chemistries of vitrimers. For example, Soman *et al.* (*17*, *29*) found a crossover from Arrhenius to non-Arrhenius behavior in both viscosity and bond rearrangement time in boronic ester ethylene vitrimers at *T*_g_/*T* ~ 0.7 to 0.8. Ge and Evans (*35*) reported a crossover of $\tau_{r}\left(T\right)$ from Arrhenius to non-Arrhenius behavior at *T*_g_/*T* ~ 0.9 in vitrimers with imine bonds. However, the estimated in this work enthalpic barrier for imine bond exchange was ~86 to 109 kJ mol^−1^ (Fig. 4C), which is more than twice higher than values found here and in other studies (*20*, *23*). We compare these data to our data in Fig. 4 (C and D) to demonstrate that they show very similar behavior. This comparison suggests that the authors of (*35*) might not have reached the final Arrhenius regime in their studies. It explains why their estimates of the enthalpic barrier were much higher and also emphasizes difficulty in estimates of the crossover region.

**Fig. 6.**
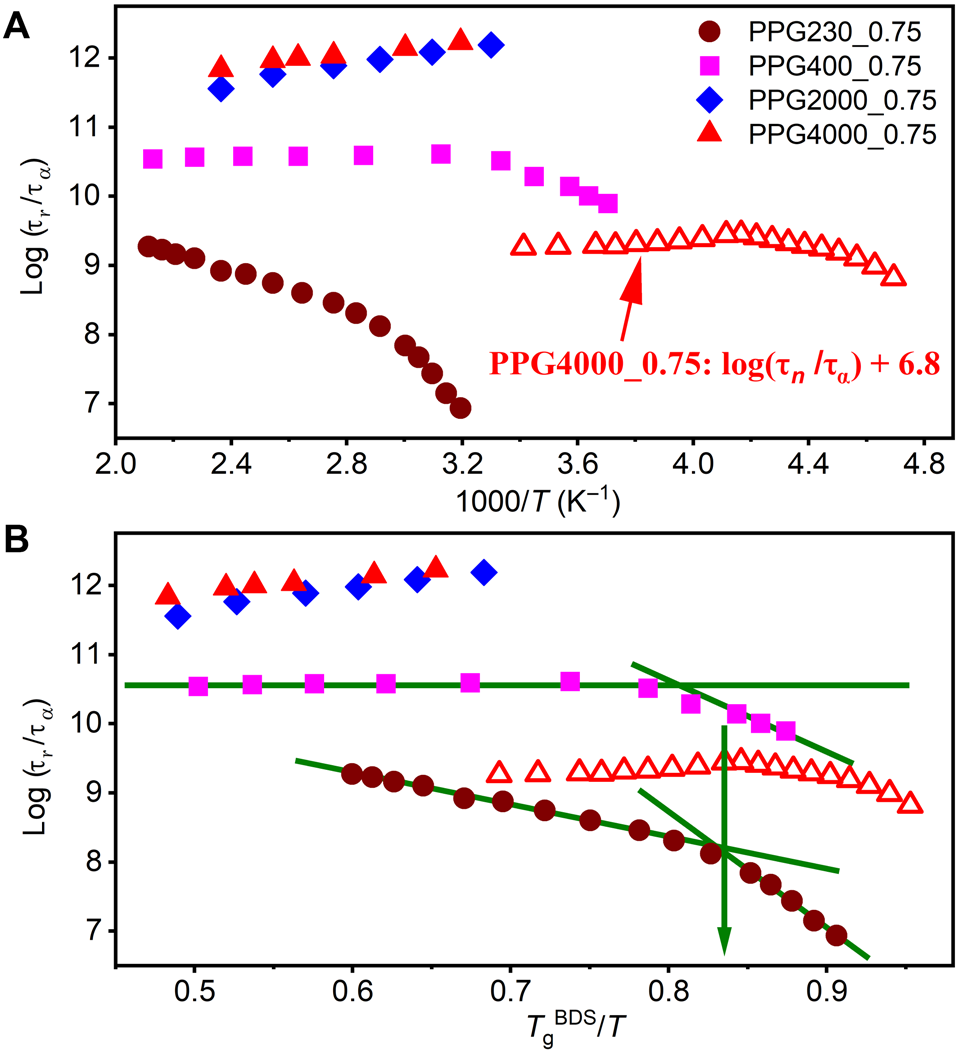
Comparison of the temperature-dependent τ_***r***_, τ_α_, and τ_*n*_. Plots of log(τ_*r*_/τ_α_) (solid) and log(τ_*n*_/τ_α_) + 6.8 (open) as a function of (**A**) 1000/*T* and (**B**) *T*_g_^BDS^/*T* for the studied vitrimers. The green lines are there to highlight the two different linear dependences, and the green arrow indicates the crossing point of the two linear regimes.

Our analysis also suggests that the temperature dependence of τ_*r*_ in chemically similar systems can be either stronger or weaker than that of τ_α_ (Fig. 6), depending on how far the experimental temperatures are from *T*_g_. We plotted log(τ_*r*_/τ_α_) versus 1000/*T* to compare the temperature dependence of τ_*r*_ and τ_α_ and to assess their relative sensitivity to inverse temperature. For PPG230_0.75, a negative slope was obtained in the whole temperature range, indicating weaker temperature variations of τ_*r*_ relative to that of τ_α_. On the other hand, a positive slope was obtained for PPG2000_0.75 and PPG4000_0.75 in the whole studied temperature range, suggesting that τ_*r*_ exhibits a stronger temperature dependence than τ_α_. Recently, Ge and Evans (*28*) performed a similar analysis of $\tau_{r}/\tau_{\alpha }$ on vitrimers with imine and boric esters bonds. For all their studied systems, $\tau_{r}/\tau_{\alpha }$versus inverse temperature showed a negative slope. Therefore, our results considerably extend their results and demonstrate that $\tau_{r}$ in vitrimers can show either a stronger or a weaker temperature dependence compared to $\tau_{\alpha }$. To understand these different trends, we used Eq. 1 to express $\frac{\tau_{r}}{\tau_{\alpha }}$ ratio

$$\frac{\tau_{r}}{\tau_{\alpha }}\approx \frac{A\text{exp}\left(\frac{E_{a}}{\mathrm{RT}}\right)+B\tau_{0}\text{exp}\left(\frac{E_{a,\text{seg}}\left(T\right)}{\mathrm{RT}}\right)}{\tau_{0}\text{exp}\left(\frac{E_{a,\text{seg}}\left(T\right)}{\mathrm{RT}}\right)}=\frac{A}{\tau_{0}}\text{exp}\left(\frac{E_{a}-E_{a,\text{seg}}\left(T\right)}{\mathrm{RT}}\right)+B$$ (5)

Equation 5 suggests that a positive or negative slope of $\text{ln}\left(\frac{\tau_{r}}{\tau_{\alpha }}\right)$ versus inverse temperature depends solely on $E_{a}-E_{a,\text{seg}}\left(T\right)$. Because $E_{a,\text{seg}}\left(T\right)$ strongly decreases with increasing temperature, Eq. 5 predicts a positive slope at certain high temperatures when $E_{a}>E_{a,\text{seg}}\left(T\right)$, showing a stronger Arrhenius-type temperature dependence of $\tau_{r}$ than $\tau_{\alpha }$ and a negative slope at low temperatures when $E_{a}<E_{a,\text{seg}}\left(T\right)$, characterizing a weaker temperature dependence of $\tau_{r}$ than $\tau_{\alpha }$. Consequently, one may expect that, for a given vitrimer, $\tau_{r}$ can show stronger, similar, or weaker temperature dependence compared to $\tau_{\alpha }$, depending on the distance of the temperature range from *T*_g_ as well as the chemically specific value of *E*_a_ for the bond rearrangement process. This helps resolve the long-standing puzzle of why some vitrimers show a stronger temperature dependence of $\tau_{r}$ than $\tau_{\alpha }$, while some others show a weaker dependence (*16*–*20*). Furthermore, for both PPG230_0.75 and PPG400_0.75, a steeper linear regime was revealed above *T*_g_/*T* ~ 0.85 (Fig. 6B). The analysis of τ_*n*_/τ_a_ versus *T*_g_/*T* revealed a similar crossover at around *T*_g_/*T* ~ 0.85, corresponding to a well-known effect of the decoupling of chain and segmental dynamics in polymers upon approaching *T*_g_ (*36*, *37*). Based on this result, we infer an active contribution of polymer chain dynamics to the bond rearrangements in studied vitrimers. The slight increase in prefactor *A* with increase in chain length (Table 1) is consistent with this idea. To account for the difference in temperature dependence of chain and segmental dynamics, the authors of (*35*) suggested using in Eq. 1 τ_α_^ν^ instead of τ_α_, where exponent ν < 1. This analysis, however, is outside the scope of the current studies.

While Eq. 1 captures qualitatively the temperature dependence of τ_*r*_, modest deviations between the experiments and the Eq. 1 prediction are visible in the crossover regime (Fig. 4, E and F). This emphasizes that the additive approach (Eq. 1) is oversimplification of the two-step process and more rigorous model should be developed. However, the largest problem of this approach is the unexpectedly large value of parameter *B*. The detailed analysis of experimental data using Eq. 1 reveals *B* ~ 10^7^ to 10^9^ in PPG230_0.75 and PPG400_0.75 (Table 1 and Fig. 4, E and 4F). Similar results were also reported recently in (*28*), where the determined *B* is around 10^6^ to 10^7^ and 10^4^ to 10^5^ in vitrimers with imine and boric ester bonds, respectively. As discussed previously in (*13*, *34*, *38*), *B* should reflect the diffusion time and the number of sticker returns to the initial partners before finding a different partner to enable stress relaxation (*13*, *24*). According to the bond lifetime renormalization model (BLRM) developed for dissociative bond exchange mechanism (*24*), the number of returns should be ~1 in the strong interaction regime when the energy barrier for bond exchange is ~30 to 40 kJ/mol (*38*). Also, the diffusion time of free ─NH_2_ groups (controlled by chain relaxation) in our systems should not be much slower than segmental relaxation time, especially in short PPG chains. Recent coarse-grained computer simulations of associative bond exchange systems (*22*) using similar approach revealed *B* ~ *N*^2^ ~ 100 (*N* is the number of beads per chain), consistent with Rouse model. However, the experimentally determined *B* values in current studies and literature are many orders of magnitude higher than this prediction.

Understanding the enormous values of *B* remains a puzzle. One possible explanation may be due to the recently proposed low steric factors or high entropic barriers, which strongly slow down bond exchange in vitrimers (*23*, *34*). The BLRM assumed that each return of a free sticker effectively results in a bond association, and, therefore, the number of returns is nearly equal to the number of bond associations/dissociations. This picture holds in physical/transient networks with the noncovalent dissociative bonds (i.e., ionic or hydrogen bonded networks) bearing unity steric factors or low entropic barriers for bond association/dissociation. However, recent works (*23*, *34*) have demonstrated that, unlike transient/physical networks, the bond exchange process in vitrimers bears extremely low steric factors *p* or very high entropic barriers Δ*S*, which strongly reduce the probability for bond exchange during a single return of a free sticker. Therefore, the total number of returns B should be much larger than the number of sticker’s returns $B^{\prime }$ defined in BLRM. We assume that these two numbers may be related by $B=\frac{B^{\prime }}{p}=B^{\prime }\text{exp}\left(-\frac{∆S}{R}\right)$, where *p* or $\text{exp}\left(\frac{∆S}{R}\right)$ quantify the probability of bond exchange during a single collision. Because $p=\text{exp}\left(\frac{∆S}{R}\right)=\frac{\tau_{0}}{A}$ based on Eq. 1, we can rewrite $B=\frac{B^{\prime }A}{\tau_{0}}$. If our assumption is correct, then *B* should be ~10^7^ in both PPG230_0.75 and PPG400_0.75 according to *A* values displayed in Table 1 and $\tau_{0}\approx 10^{-13}$ s. While this *B* value agrees with estimates obtained for PPG230_0.75, it is ~2 orders of magnitude lower than that determined for PPG400_0.75 (Table 1). Applying the same assumptions to the vitrimers data reported in (*28*) results in *B* values ~1 to 2 and ~3 to 5 orders of magnitude higher than the values predicted by Eq. 1 for systems containing boric ester and imine bonds, respectively. Thus, although the proposed picture suggests some qualitative role of steric factors and entropic barriers in this unexpectedly large value of parameter *B*, it fails to describe the existing data on a quantitative level.

An alternative explanation can be based on the recent idea proposed in (*22*) about mean-squared displacements (MSDs) of functional groups required for bond rearrangements, $\langle r_{\text{bond}}^{2}\rangle$. If it is smaller than equilibrium thermal fluctuations of functional groups before segmental relaxation $\langle r^{2}\left(T\right)\rangle$ (e.g., vibrational plus rattling in a cage), then the bond rearrangement does not require segmental motion. In the case of these simulations, $\langle r_{\text{bond}}^{2}\rangle$ was always smaller than $\langle r^{2}\left(T\right)\rangle$ (*22*), and the estimated *B* value was consistent with the expected functional groups diffusion (see above). Following this idea, we can assume that, at high temperatures, thermal fluctuations are larger than required $\langle r_{\text{bond}}^{2}\rangle$, leading to bond rearrangements with no remarkable dependence on segmental dynamics. However, the amplitude of thermal fluctuations decreases strongly with cooling. It has been shown experimentally (*39*, *40*), computationally (*41*), and rationalized theoretically (*42*) that, in rough approximation, $\text{log}\left[\frac{\tau_{\alpha }\left(T\right)}{\tau_{0}}\right]\propto 1/\langle r^{2}\left(T\right)\rangle$ (here, $\langle r^{2}\left(T\right)\rangle$ presents MSD on picosecond timescale). Thus, at some τ_α_(*T*), $\langle r^{2}\left(T\right)\rangle$ becomes smaller than that required for bond rearrangements. In that case, segmental relaxation would be involved to reach the required MSD. This idea is consistent with the crossover from Arrhenius to super-Arrhenius behavior to be at approximately the same value of *T*_g_/*T* (Fig. 4B) in our systems because they have the same behavior of τ_α_(*T*/*T*_g_). However, it might be different for polymers with different fragility or for different dynamic bonds that might require different $\langle r_{\text{bond}}^{2}\rangle$. Computer simulations with varying $\langle r_{\text{bond}}^{2}\rangle$ can test this hypothesis. In any case, we want to emphasize that a quantitative understanding of the parameter *B* remains a challenge and it is obviously much larger than the value expected in the BLRM.

In summary, our studies indicated that bond rearrangement in vitrimers is controlled by a combination of bond exchange kinetics and segmental diffusion, leading to two regimes: (i) Arrhenius behavior at high temperatures (far above *T*_g_) controlled by bond exchange kinetics and (ii) super-Arrhenius behavior at low temperatures (near *T*_g_) where segmental motions also become important. The crossover between these two regimes occurs at temperatures where *T*_g_/*T* ~ 0.7 in the now studied vitrimers, where segmental mobility begins to influence the apparent activation energy barrier of bond rearrangements. Our studies also demonstrated that τ_*r*_ can exhibit either a stronger or weaker temperature dependence than segmental relaxation time, depending on the distance of the studied temperature regime from *T*_g_ and the bond exchange enthalpic barrier *E*_a_. These results offer experimental support for the recently proposed two-timescale approach to describe τ_*r*_ in vitrimers (*19*, *21*). However, they also revealed quantitative problems of this oversimplified description. Detailed analysis of the experimental data based on this framework reveals an unexpectedly large coefficient *B* relating τ_*r*_ and τ_α_. The origin of this large value remains unknown and requires further studies including computer simulations.

Moreover, we confirm again that a substantial entropic barrier, arising from strict geometric requirements for successful associative bond exchange strongly slows down bond exchange in vitrimers. This entropic barrier should depend on the specific chemistry of dynamic covalent bonds, and a fundamental understanding of this entropic contribution will be important for the rational design of vitrimers with tailored macroscopic viscoelastic properties. Our study further indicates the potential role of chain relaxation in bond rearrangements in vitrimers, although the fundamental mechanism on a quantitative level remains unclear.

## MATERIALS AND METHODS

### Synthesis of vitrimers

We synthesized model vitrimers via the reaction between linear amino-terminated PPG purchased from Sigma-Aldrich and BTA (purity, 98%) purchased from Tokyo Chemical Industry. All samples were used as received. Amino-terminated PPG samples with varying molecular weights (*M*_*n*_ = 230, 400, 2000, and 4000 g/mol) were cross-linked by BTA bearing tri-aldehyde groups (Fig. 1A), with a molar ratio of 0.75 between aldehyde and amine groups. Our previous work (*23*) has confirmed that a well-defined network will form with this ratio. For the synthesis, 2 g of PPG was initially dissolved in 10 ml of tetrahydrofuran (THF; 99.9%, anhydrous, inhibitor-free, Sigma-Aldrich). Under stirring, this solution was added dropwise to BTA solutions in THF, with concentrations adjusted to ensure a molar ratio 0.75 between aldehyde and amine groups. This mixture was reacted at 40°C under nitrogen protection for 18 to 30 hours. After evaporating the THF using rotary evaporation, the solution was poured into a Teflon dish and moved into a vacuum oven to further evaporate the solvent at 40°C for 24 hours and then further dried at 85°C for 48 hours. The synthesized vitrimers and corresponding PPG precursors are named PPG*x*_0.75 and PPG*x*, respectively, where *x* denotes the molecular weight of the PPG precursors.

### Raman spectroscopy, SAXS, TGA, and DSC

The chemical structures of the synthesized samples were verified using a T64000 Raman spectrometer from Horiba Jobin Yvon. The experiments were performed using a backscattering geometry and laser with a wavelength of 660 nm at room temperature. To minimize moisture effects, the Raman measurements were conducted immediately after removing the samples from the oven. X-ray measurements were performed using a Xenocs Xeuss 3.0 SAXS instrument with monochromatic Cu Kα high-intensity x-ray beam with wavelength of 1.54 Å. The measurements were conducted at room temperature and under vacuum conditions. The exposure time is 600 s. The thermal properties (i.e., glass transition and thermal stability) of the samples were evaluated with DSC2500 (TA Instruments) and TGAQ50. The DSC measurements were performed under a helium protective gas flow of 50 ml/min. For each sample, three cooling-heating cycles were performed with a running rate of 10 K/min. The TGA measurements were performed with a running rate of 10 K/min under N_2_ gas flow of 50 ml/min.

### Broadband dielectric spectroscopy

Dielectric measurements in the frequency range from 10^−2^ to 10^6^ Hz were performed using a Novocontrol GMBH Alfa impedance analyzer. These measurements were performed in a parallel plate dielectric cell made of sapphire and invar steel with an electrode diameter of 10 mm and a capacitance of ∼3.45 pF with an electrode separation of ∼0.25 mm. Agilent 4291B impedance analyzer connected with the Novocontrol system was used for the high-frequency measurements (10^6^ to 10^9^ Hz) with the sample placed between two gold-plated electrodes with diameter of 10 mm and gap of ∼0.20 mm. During the dielectric measurements, the sample was equilibrated for 10 min at each temperature to reach thermal stabilization within 0.2 K. Before measurements, the sample was dried in broadband dielectric spectroscopy (BDS) system at 423 K for 2 hours. An ac electric voltage of 1.5 V was applied for all measurements.

### SAOS rheology

Rheological measurements were performed using strain-controlled mode of an AR2000ex rheometer (TA Instruments). SAOS experiments were performed to explore the linear viscoelasticity of studied samples in angular frequency range from 10^−3^ to 10^2^ rad/s with an applied strain of 3%. Eight-millimeter-diameter parallel plates were used for all measurements. The samples were equilibrated before each measurement to prevent temperature deviations larger than 0.2 K, and the experiments were conducted under an N_2_ flow. Before measurements, the sample was dried in the rheometer at 423 K for 2 hours.
